# Changes in the Gut Bacteria Composition of Healthy Men with the Same Nutritional Profile Undergoing 10-Week Aerobic Exercise Training: A Randomized Controlled Trial

**DOI:** 10.3390/nu13082839

**Published:** 2021-08-18

**Authors:** Ayane S. Resende, Geovana S. F. Leite, Antonio H. Lancha Junior

**Affiliations:** 1 Health Sciences Graduate Program, Federal University of Sergipe, São Cristovão 49100-000, SE, Brazil; 2 Laboratory of Nutrition and Metabolism Applied to Motor Activity, School of Physical Education and Sports, University of Sao Paulo, São Paulo 05508-030, SP, Brazil; geovana.leite@usp.br; 3 Laboratory of Clinical Investigation: Experimental Surgery (LIM/26), Clinics’ Hospital of Medical School, University of Sao Paulo, São Paulo 01246-903, SP, Brazil; lanchajunior@gmail.com

**Keywords:** gut microbiota, human, exercise, sedentary behavior, VO_2_max, body mass index, diet

## Abstract

Nutrient consumption and body mass index (BMI) are closely related to the gut microbiota, and exercise effects on gut bacteria composition may be related to those variables. Thus, we aimed to investigate the effect of 10-week moderate aerobic exercise on the cardiorespiratory fitness and gut bacteria composition of non-obese men with the same nutritional profile. Twenty-four previously sedentary men (age 25.18 [SD 4.66] years, BMI 24.5 [SD 3.72] kg/m^2^) were randomly assigned into Control (CG; *n* = 12) or Exercise Groups (EG; *n* = 12). Body composition, cardiorespiratory parameters, blood markers, dietary habits and gut bacteria composition were evaluated. EG performed 150 min per week of supervised moderate (60–65% of VO_2_peak) aerobic exercise, while CG maintained their daily routine. The V4 16S rRNA gene was sequenced and treated using QIIME software. Only EG demonstrated marked improvements in cardiorespiratory fitness (VO_2_peak, *p* < 0.05; Effect Size = 0.971) without changes in other gut bacteria-affecting variables. Exercise did not promote clustering based on diversity indices (*p* > 0.05), although significant variations in an unclassified genus from *Clostridiales* order and in *Streptococcus* genus were observed (*p* < 0.05). Moreover, α-diversity was correlated with VO_2_peak (Pearson’s R: 0.47; R^2^ 0.23: 95%CI: 0.09 to 0.74, *p* = 0.02) and BMI (Pearson’s R: −0.50; R^2^ 0.25: 95%CI: −0.75 to −0.12, *p* = 0.01). *Roseburia*, *Sutterella* and *Odoribacter* genera were associated with VO_2_peak, while *Desulfovibrio* and *Faecalibacterium* genera were associated with body composition (*p* < 0.05). Our study indicates that aerobic exercise at moderate intensity improved VO_2_peak and affected gut bacteria composition of non-obese men who maintained a balanced consumption of nutrients.

## 1. Introduction

Sedentary behavior is the fourth main cause of major Noncommunicable Diseases (NCD) such as obesity, diabetes, cardiovascular disease and colon cancer [1,2]. Approximately one-third of adults across the world’s population does not achieve the minimum exercise frequency recommended by the World Health Organization (WHO) [3], i.e., 150 min per week of moderate aerobic and/or anaerobic exercise or 75 min of vigorous exercise [4]. Consistent evidence has shown that exercise promotes several health benefits, even in young and healthy individuals [2,5], and can also influence gut microbiota composition [6]. The human gastrointestinal tract harbors a dynamic microbial ecosystem which contributes to the host’s metabolic and immune functions. In fact, bacteria from the human gut microbiota play an important role in the development of NCD [7,8,9]. Le chatelier et al. [10] and Liu et al. [11] observed greater body mass index (BMI), adiposity, insulin resistance, dyslipidemia and a more pronounced inflammatory status in individuals who also presented significantly reduced gut bacteria diversity. Even in healthy and normal weight subjects, Sket et al. [12,13] demonstrated that 21 days of predominantly sedentary behavior promoted both functional and compositional modifications to the gut microbiota that were associated with a reduction in intestinal transit time and an enhancement in the concentration of secondary bile acids and neurotoxins within stool samples. Hence, science has progressed towards environmental approaches that positively influence human gut microbiota and health, such as exercise [14,15].

Eight weeks of moderate aerobic exercise improves cardiorespiratory fitness by increasing oxygen uptake (VO_2_peak) [16] and enhances resilience against inflammatory stressors, such as lipopolysaccharides (LPS) [17]. Moderate aerobic exercise affects the intestinal system mainly through gut immune function [18]; gut barrier integrity through tight junction proteins expression [19] and IgA production [20]; hypothalamic–pituitary–adrenal (HPA) axis stimulation which, in turn, affects enteric nervous system and intestinal transit time, as well as gut motility, intestinal pH and gut hormones release [20]; and bile acids metabolism within enterohepatic circulation [21]. These exercise-induced intestinal adaptations affect the gut environment in a way that may select the surviving microorganisms, leading to alterations to gut microbiota composition [14,20,22].

Longitudinal studies have demonstrated that short to moderate term aerobic exercise induces gut bacteria composition alterations in previously sedentary adults [23,24,25,26], although these studies have included non-healthy individuals. Furthermore, each study demonstrated different results, which were strongly dependent on other variables, such as BMI status [24,26], age range [23] and diet [25]. For instance, Allen et al. [24] observed that *Faecalibacterium* genus increased in lean subjects and decreased in obese subjects after six weeks of moderate aerobic training. In contrast, *Bacteroides* genus decreased in lean subjects and increased in obese ones after training. On the other hand, Munukka et al. [26] did not observe the same alterations in *Faecalibacterium* and *Bacteroides* genera. Studies performed on athletes have also demonstrated varied results [6] which are associated with their respective lifestyles, including diet habits and training level, as well as differences between males and females that can affect the gut microbiota, as suggested by Estaki et al. [27]. These studies have shown the importance of the composition of gut bacteria on human health and that aerobic exercise is capable of modulating this composition. However, our current understanding of the effect of exercise on human gut microbiota composition is influenced by diseases, obesity, dietary interventions and different experimental designs. In order to improve our recommendations in clinical practice, especially for sedentary people, we have to analyze exercise effects in a more controlled scenario.

Here, we show results from a controlled and randomized study that investigated the effect of a 10-week period of aerobic exercise on the gut bacteria composition of apparently healthy and sedentary young men. Our hypothesis was that 10 weeks of exercise training would be efficient at improving aerobic fitness (VO_2_peak) and at modifying bacterial taxa diversity and abundances.

## 2. Materials and Methods

### 2.1. Subjects

**Figure 1:**
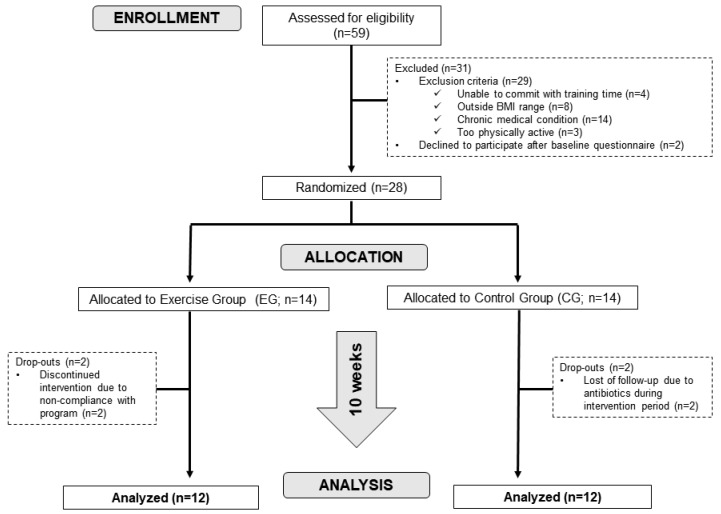
CONSORT Flow Diagram.

Healthy Brazilian men aged 20–45 years were recruited at the University of São Paulo campus (Sao Paulo, SP, Brazil) to participate in a randomized parallel-arms con-trolled study. Exclusion criteria were: (1) underweight (BMI < 18.5 kg/m^2^) or obese (BMI ≥ 30 kg/m^2^); (2) engaged in exercise training or performing more than 150 min of any physical activity per week (according to the International Physical Activity Questionnaire; IPAQ); (3) following a vegan or other restrictive diet, such as intermittent fasting or a low carbohydrate diet; (4) compulsive habit of consuming alcohol and/or smoking [28]; (5) reported diagnosis of major gastrointestinal disorders (food intolerance or allergy, celiac disease, inflammatory bowel disease, irritable bowel syndrome, chronic constipation), autoimmune disease, any non-communicable diseases such as cardiovascular diseases, hypertension, cancer history, eating disorders, hypothyroidism or other metabolic or neurological disease, and/or musculoskeletal disorders that could preclude the ability to perform training and testing; and (6) taking oral or topical antibiotics, laxatives, nutritional supplementation, pre- or probiotics within six months prior to the sampling or during the study. All volunteers were residents of Sao Paulo city and were graduate students, post-graduate students or public servants at the University of Sao Paulo. They also confirmed that they had maintained a consistent lifestyle (sedentary behavior, diet and body weight) in the last 6 months. Figure 1 shows the flow diagram. All procedures of the present study were conducted according to the principles of the Sixth Declaration of Helsinki. Written informed consent was obtained from all participants before their enrollment into the study.

### 2.2. Study Design and Exercise Training Protocol

**Figure 2:**
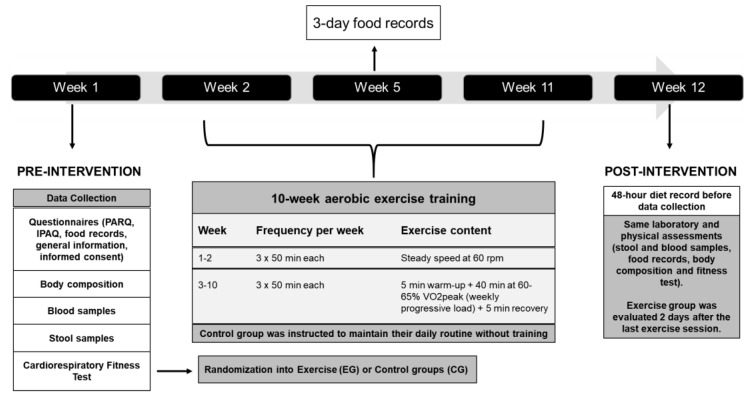
Study design, measurements and exercise protocol. Legend: IPAQ: International Physical Activity Questionnaire; PARQ: Physical Activity Readiness Questionnaire; VO_2_peak: peak oxygen consumption; rpm: rotations per minute.

The study design lasted 12 weeks for each participant, as presented in Figure 2. At the baseline (Pre), questionnaires were applied in order to evaluate inclusion and exclusion criteria. Fecal and blood samples were also collected and cardiorespiratory fitness was determined by an incremental maximum test performed in cycle ergometers, as described in the next section. After the baseline measurements, eligible participants were matched for age, VO_2_peak and BMI, and then randomly assigned into two groups, Exercise Group (EG) or Control Group (CG). The randomization process was performed by a third person and participants in both groups were paired according to their VO_2_peak. Supplemental Table S1 shows the subjects’ distribution between groups after randomization. The volunteers from EG carried out the exercise training program for 10 weeks, while the subjects from CG did not engage in any exercise program during the same period. All participants were weekly instructed to maintain their lifestyle and diet habits during the study. In particular, CG subjects received instructions weekly by phone in order to maintain their adherence. As shown in Figure 2, three-day food records were collected from both groups during week 5. After the 10-week period, the same procedures used to measure the baseline (Pre) were reproduced during the last week of data collection (Post), and to this end all participants were instructed to consume the same food recorded from the baseline measurements during the 48 h prior to stool collection at the post-experiment measurements (Figure 2). Photos and food records were requested in order to ensure compliance with this instruction. Additionally, all participants were asked to refrain from the consumption of alcohol and medication for at least 24 h prior to measurements.

The exercise training protocol was designed to achieve the minimum recommendations outlined by the WHO [4,29]. All training sessions were carried out on a cycle ergometer (Bicycle 2600 Electromagnetic Movement). Three supervised training sessions per week on non-consecutive days, lasting 50 min each, were performed over 10 weeks (Figure 2). Our exercise protocol was designed to improve the cardiorespiratory fitness of previously sedentary men over a sufficient time period to affect gut bacteria composition. Based on previous studies, 6–8 weeks of continuous moderate aerobic training could achieve these results [15,16]. Thus, we chose 10 weeks of exercise training in order to analyze the chronic effect of regular training in apparently healthy men. EG subjects usually exercised during the same shift of the day. The workload (W) level corresponding to 60–65% relative intensity of VO_2_peak of each subject was monitored during each session. During the first and second weeks of exercise intervention, 50 min of steady-state cycling at the lower intensity of the range (60% of VO_2_peak) was performed as participants had undergone no exercise training for at least one year previous to this study. Over the next weeks, the resistance of the cycle ergometer was progressively increased by adding a load between 15–25 W in order to maintain training intensity between 60–65% of the individual’s VO_2_peak throughout the 10 weeks of exercise training. The frequency of workload addition was specific to each volunteer and was considered the load at which the subject was able to maintain 60 rpm over 40 min. Thus, workloads could be adjusted in the middle of training in order to maintain the training intensity. This intensity was monitored using HR and Borg’s scale [30]. The Control Group remained untrained for 10 weeks (Figure 2) [16].

### 2.3. Laboratory and Physical Measurements

#### 2.3.1. Cardiorespiratory Fitness Testing

At the baseline, before the cardiorespiratory fitness test, participants completed a physical activity readiness questionnaire (PAR-Q) developed by the American College of Sports Medicine to ensure safe participation [31]. Subsequently, an incremental maximum exercise test was carried out using a bicycle ergometer (Biotec 2100, Cefise, Nova Odessa, Brazil) with open-circuit spirometry attached to a Cortex gas analyzer (MetaLyzer^®^ 3B) in order to determine peak oxygen uptake (VO_2_peak) and associated peak power output. These exercise tests were scheduled either for the morning (9 a.m.–11 a.m.) or afternoon (2 p.m.–5 p.m.). All tests were carried out in an air-conditioned room with temperature of approximately 23–24 °C and relative humidity ≥50%. Volunteers were instructed to eat a meal two hours before the exercise test, maintaining their food habits. The test started at 20 watts (W) for three minutes to warm-up. The workload was then set at 50 W and the volunteers were instructed to maintain a pedal speed of 60 RPM. At each minute of the test, it was increased by a load equivalent to 25 W and these increments would be carried out until the end of the test. Subjects’ perception of exertion was monitored using Borg’s 6–20-point scale at the end of each stage [30] and HR was monitored throughout the test (Polar, Kempele, Finland). Criteria for achieving VO_2_peak were the following: (a) voluntary exhaustion; (b) respiratory exchange ratio (RER) > 1.15; (c) plateau in VO_2_; and/or (d) RPM falling below 50. At the end of the test, the volunteers cycled for another four minutes with a minimal load (20 W) as a cool down. This protocol was adapted from studies carried out by Estaki et al. [27] and Matsuo et al. [16] and was based on fitness test guidelines [29]. HR peak and VO_2_peak were determined as the mean of the final 20 s of the test. Afterwards, subjects were classified according to their VO_2_peak into low (<37 mL·kg^−1^·min^−1^), average (38–44 mL·kg^−1^·min^−1^) or high (>45 mL·kg^−1^·min^−1^) groups based on current references for men between 20–29 years [27,29]. These same conditions of the incremental maximum exercise test were reproduced 48 h after the last exercise session for post-intervention evaluation of cardiorespiratory fitness parameters (Figure 2).

#### 2.3.2. Dietary Data Collection

Food habits were evaluated from a 48 h food record, a food frequency questionnaire (FFQ) and a three-day food record (2 weekdays and 1 weekend day). The 48 h food record was collected during week 1 (Pre) in order to analyze total energy intake, macronutrients, fiber, cholesterol and water, and was also important for reproducing the same diet during the 48 h prior to stool collection during week 12 (Post), as shown in Figure 2. The ELSA-BRAZIL FFQ [32] was applied at the baseline to evaluate food groups consumption over the six months prior to the trial (bread, cereals and tubers, fruits, vegetables, legumes, milk, yogurt and cheese, meat, fish, eggs and sweets). One food group is composed by foods that have similar proportions of nutrients and chemical characteristics. The ELSA-BRAZIL FFQ reduced form comprises 76 items and was validated for the Brazilian adult population [32]. The three-day food record was collected during week 5 in order to evaluate the maintenance of food habits. All food questionnaires were applied in both groups using photographs of all food and portions investigated. All data were analyzed with AvaNutri^TM^ software (Rio de Janeiro, Brazil), which uses the Brazilian tables of nutritional composition as a database for the calculation of nutrients [33].

#### 2.3.3. Body Composition Assessment

Body height (cm) was measured by using a wall-fixed measuring device. Weight (kg) and body composition, such as total body fat mass (%) and free-fat mass (%), were assessed via plethysmography of the whole body (air displacement plethysmography, BOD POD^®^ body composition system; Life Measurement Instruments, CA, USA) at the Pre and Post interventions [34]. BMI was calculated as: $\mathrm{weight}\;\left(\mathrm{kg}\right)\;÷\;\mathrm{height}\;{\left(m\right)}^{2}$. Categorical classifications for our purposes were based on WHO [35]; therefore, the trial included eutrophic (BMI: 18.5 kg/m^2^–24.9 kg/m^2^) and overweight (BMI: 25 kg/m^2^–29.9 kg/m^2^) men.

#### 2.3.4. Blood Samples

Blood samples were taken from each volunteer by venipuncture in the morning (7–10 a.m.) after overnight 12 h fasting. The plasma was separated by centrifugation for 10 min at 3000 rpm and 4 °C (Universal 32R, Hettich, Buckinghamshire, UK) and stored at −80 °C until analyses. Fasting glucose, total cholesterol, high density lipoprotein-cholesterol (HDL-c), low density lipoprotein-cholesterol (LDL-c) and triglycerides were measured by enzymatic colorimetric assay using commercial kits (Biotecnica, Minas Gerais, Brazil).

#### 2.3.5. Fecal Samples

At week 1 of the study design, participants were provided with a sterilized stool collection kit to collect a stool sample in their home. The stool kit contained a 50 mL stool collector, a spatula and a sanitary seat cover that allowed the volunteers to evacuate in a common sanitary seat at their home and then collect the stool sample without contaminating it with toilet water (ColOFF^®^, São Paulo, Brazil). A pair of disposable gloves were also delivered for use at the time of collection to minimize any contamination. They were instructed to immediately freeze the samples at −4 °C after stool collection, and all volunteers delivered this frozen stool sample within 24 h of collection. Styrofoam was used to store the collector-contained stool samples at the time of transportation to the laboratory. As soon as the stool samples were delivered to the laboratory, they were mixed and weighed inside a sterilized Laminar Flow Chapel (Veco do Brasil, Campinas, Brazil). Aliquots were immediately stored at −80 °C until analysis [36]. At the post intervention, the stool samples from EG were collected 48 h after the last exercise session to minimize the acute effect of the last training session.

### 2.4. DNA Extraction, 16S rRNA Gene Sequencing and Bioinformatics

The gut bacteria composition analysis was performed from a total of 48 stool samples (pre-intervention: EG = 12, CG = 12; post intervention: EG = 12, CG = 12). First, total bacterial DNA was extracted from feces using the QIAamp fast DNA stool mini kit (Qiagen, Valencia, CA, USA) strictly following the manufacturer’s recommendations. After extraction, DNA was quantified by spectrophotometry (NanoDrop-ND 2000). The aliquots containing the extracted bacterial DNA were immediately transported in dry ice-containing styrofoam to the BPI Genotyping Laboratory, which was responsible for 16S rRNA gene sequencing (Botucatu, SP, Brazil). Total DNA extracted was analyzed using quality agarose gel stained with Red Gel (Uniscience) and visualized under ultraviolet light.

Next, sequence libraries were prepared according to the Illumina MiSeq system instructions (Illumina, San Diego, CA, USA). Variable region V4 of the 16S rRNA gene was amplified. All procedures involved in this analysis followed the recommendations of the Illumina platform and the same flow described by Caporaso et al. [37]. Briefly, amplification reactions of the 16S rRNA V4 were performed through real-time quantitative polymerase chain reaction (RT-PCR), resulting in a final volume of 20 μL containing 10 μL of GoTaq^®^ Colorless Master Mix 2x (Promega, USL), 0.3 μM forward oligonucleotide and 0.3 μM reverse oligonucleotide, 30 ng genomic DNA, and sterile ultrapure water sufficient to make up 20 μL. For the amplification, primers 515F and 806R were used, respectively:

- 16S–V4 Forward 5′–TCGTCGGCAGCGTCAGATGTGTATAAGAGACAGGTGCCAGCMGCCGCGGTAA–3′
- 16S–V4 Reverse 5′–GTCTCGTGGGCTCGGAGATGTGTATAAGAGACAGGGACTACHVGGGGTWTCTAAT–3′

Amplification reactions were conducted on the Veriti™ Thermal Cycler (Applied Biosystems, Foster City, CA, USA) and then subjected to purification steps using an Agencourt AMPure XP magnetic bead (Beckman Coulter) according to the manufacturer’s instructions. An indexing step was performed for organization and identification of the readings, in which indexers were inserted in the common adapters (8-bp barcodes specific to each sample). This reaction was performed by PCR following the Nextera XT Index kit protocol (Illumina). The indexed readings were subjected to purification steps, also using the Agencourt AMPure XP magnetic bead (Beckman Coulter), for the removal of very small fragments from the total population of primers molecules and residues. Quantification was performed via the RT-PCR methodology using QuantStudio 3 Real Time (Applied Biosystems, Foster City, CA, USA) and a Kit KAPA-KK4824 (Library Quantification Kit-Illumina/Universal) thermal cycler, according to the manufacturer’s instructions. An equimolar pool of DNA was generated by normalizing all samples at 2 nM for sequencing, which was conducted using the Illumina MiSeq next-generation sequencing system (Illumina^®^ Sequencing). The samples were sequenced from both ends (paired-ends, 2 × 150 bp).

The sequencing coverage was around 156,250 readings per sample. A total of 4,044,921 original fastq sequences were obtained after sequencing and processed using QIIME software (Quantitative Insights Into Microbial Ecology) pipeline 2 version 2020.2. The workflow and steps chosen for the treatment of these sequences were those described by Boylen et al. [38] in accordance with the QIIME2 tools and recommendations detailed on their website (https://docs.qiime2.ogr (accessed on 1 December 2019)). The sequences used built-in DADA2 software package v1.6 [39] to filter contaminants, denoising and chimeras, after which the readings from each end (2 × 150 bp) were merged, considering at least 6 base pairs (bp) of overlap. After quality filtering, operational taxonomic units (OTUs) were identified and used to build a phylogenetic tree. The proportion of each OTU in each sample was used for taxonomic assignment and diversity analyses. The sequences were contrasted against Greengenes version 13.5 for taxonomic analysis, with minimum confidence threshold of 80% [40]. The BIOM file was converted to generate a table with taxonomic consensus up to the genus level and, from this, statistical procedures were performed. After the bioinformatics, a total of 140,500 16S rRNA high quality sequencing reads were obtained from the 48 stool samples. The median read count per sample was 2927 [1811; 4545] and 2121 different OTUs were detected from those final sequences (Supplemental Table S3). Alpha- and β-diversity were obtained in QIIME2 software. Alpha diversity was calculated using the Shannon, Observed Species and Pielou evenness indices. Rarefaction curves based on Shannon index and observed species were virtually saturated with 1667 sequences, suggesting sufficient sequencing depth (Supplemental Figure S2). Beta diversity was computed using UniFrac weighted and unweighted distances and a Bray–Curtis dissimilarity matrix. These matrices generated the Principal Coordinates Analysis (PCoA) plots and were visualized using EMPeror [41]. QIIME2’s view tool was used to visualize artifacts and graphs.

### 2.5. Statistical Analysis

The sample size was calculated according to Matsuo et al. [16], which showed an increase of 14.7% in VO_2_peak in previously sedentary men after an eight-week (three days a week) supervised exercise intervention. To accommodate for dropouts and technical issues during the study, an extra 30% was added, resulting in approximately 10 subjects per group. This calculation was performed using BioEstat software version 5.0 with an alpha of 5% and a beta-power of 95% (two-tailed). No sample size calculations were done specifically on the gut microbiota measures, as there were no similar previous studies at the time of the study planning.

Data from body composition, blood samples, cardiorespiratory fitness test, dietary questionnaires and gut bacteria composition (number of sequences, Shannon index, observed species, Pielou evenness, Firmicutes/Bacteroidetes ratio and relative abundance of phyla and genera) were tested for their normality using the Shapiro–Wilk test. When possible, variables presenting a non-Gaussian distribution were transformed to their natural logarithms. Normally distributed data were represented as mean ± standard deviation (SD) or as mean and 95% confidence interval (CI95%) or, for non-normally distributed data, as median and their minimum and maximum ranges. Differences between Groups at the pre-intervention (Pre) were tested using Student’s *t*-test (two tails and unpaired samples) with Welch’s correction or the corresponding non-parametric test. In order to test the differences after intervention, two-way ANOVA with repeated measures was used for normally distributed data, with “Group” and “Time” as factors. In the case of a significant F value, the Tukey post-hoc test was applied. Effect size (ES) was calculated using Cohen’s coefficient. Qualitative descriptors for the interpretation of ES were designated as follows: <0.2, trivial effect; 0.2–0.39, small effect; 0.40–0.75, moderate effect; >0.75, large effect. Regarding gut bacteria, PERMANOVA (Permutational Multivariate Analysis of Variance) test with 999 random permutations was applied in the QIIME2 environment to determine if there were statistical differences within the β-diversity indices over time between study Groups. Subsequently, relative abundance of each OTU in each sample was calculated in order to determine the proportion of sequences. Relative abundance (%) was determined by the equation: $\left[\%\mathrm{abundance}\;=\;\left(\mathrm{number}\;\mathrm{of}\;\mathrm{sequences}\;\mathrm{of}\;\mathrm{each}\;\mathrm{OTU}\;\times 100\right)\;÷\;\mathrm{total}\;\mathrm{sequences}\;\mathrm{per}\;\mathrm{sample}\right]$. For further analysis, only genera detected in ≥25% of the 48 samples were considered. In order to investigate differences in the variation of each genus from the pre- to post-intervention between Groups, the delta value was calculated for each subject. The delta (∆) was obtained from the equation: %genus POST−%genus PRE. This method was chosen with the goal of preserving intragroup variations over the 10-week period [42]. As the majority of taxa did not present a Gaussian distribution, the Mann–Whitney test for independent measures better fit these assumptions and was applied to compare ∆EG and ∆CG for each genus (Supplemental Table S7). To identify statistically relevant species, Analysis of Composition of Microbiomes (ANCOM) was performed [43], considering Group and time. In addition, to explore associations between the exploratory variables and microbial composition data, Pearson’s or Spearman’s correlation coefficient was calculated [44].

Statistical analyses of exploratory variables (body composition, blood parameters, VO_2_peak and Wpeak and dietary data), delta differences and correlations were conducted using GraphPad Prism software version 9.0.2. For all analyses, statistical differences were considered when *p* < 0.05, following adjustments for potential confounders (BMI, fat mass, fasting glucose, triglycerides, carbohydrate and protein intake). For multiple comparisons the False Discovery Rate method of Benjamini and Hochberg was applied.

## 3. Results

### 3.1. Aerobic Exercise Increased Cardiorespiratory Fitness without Changing Body Composition or Plasma Metabolic Parameters

At baseline, EG and CG shared similar clinical and anthropometric characteristics (*p* > 0.05, Table 1). All study participants were of high educational level and none of the volunteers reported having difficulties with defecation or gastrointestinal symptoms, such as diarrhea, nausea or abdominal pain, during the study design. Following the intervention period, only EG demonstrated marked improvements in cardiorespiratory fitness parameters (*p* < 0.05, Table 1, Supplemental Figure S1).

Compliance with the prescribed exercise program was high (100%); that is, all EG subjects completed 30 supervised training sessions according to our study protocol. Statistical analysis revealed a significant intragroup effect in EG with a mean increase of 16.48% in VO_2_peak (*p* = 0.001, ES = 0.971) and 19.99% in Wpeak (*p* < 0.001, ES = 0.976). Anaerobic threshold 1 (AT1) also significantly improved in EG (*p* = 0.033, ES = 0.58) after 10 weeks of exercise training, while the CG subjects maintained their cardiorespiratory parameters. Conversely, changes were not observed in body composition and plasma metabolic parameters in either EG or CG (*p* > 0.05, Table 1). There were no changes in evacuation frequency reported by volunteers between pre- and post-intervention.

Furthermore, all volunteers from both groups presented very similar food habits at pre-intervention and habitual consumption of all food groups in the last 6 months (*p* > 0.05; Supplemental Table S2). These food habits contributed to a daily mean consumption of 51.9% (±8.0%) carbohydrates, 17.4% (±5.2%) protein and 30.6% (±5.9%) total fat. The dietary habits were strictly maintained during the study, and no significant changes were detected in total energy, food groups or gut microbiota-affecting nutrients during and after the 10-week period (*p* > 0.05, Supplemental Table S2). Due to the success of our study design in improving cardiorespiratory fitness without changing eating habits, body composition and plasma metabolic parameters, it was possible to preserve the exercise effect on the gut bacteria composition. Subsequently, we analyzed stool samples from age-, VO_2_peak- and BMI-matched participants of CG and EG.

### 3.2. Effect of Exercise-Induced Improvements on Gut Bacteria Diversity

Considering that α- and β-diversity are important measures of the human gut bacteria structure, which are closely related to subjects’ health status, our hypothesis was that exercise-induced cardiorespiratory fitness improvements would significantly differentiate trained from sedentary individuals. However, two-way ANOVA with repeated measures did not detect significant differences as effects of exercise on any α-diversity index—neither between Groups nor within groups between pre- and post-intervention (Supplemental Figure S2 and Table S4).

**Figure 3:**
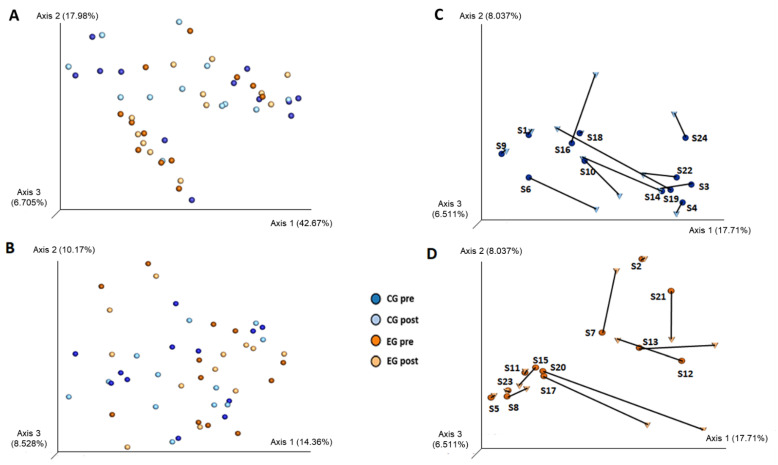
Principal coordinates analysis (PCoA) of gut bacteria composition. At the left of the figure, PCoA plots were constructed using (**A**) UniFrac weighted and (**B**) UniFrac unweighted indices, which were based on the 1667 microbial sequences from the rarefaction curve. Each point in the PCoA plot represents one individual, which is colored to represent the Group and Time measured. At the right of the figure, intraindividual variations of microbial community structure within (**C**) Control and (**D**) Exercise Groups using a Bray–Curtis dissimilarity PCoA plot at the genus level. There was no difference between Pre- and Post-intervention in either Group (PERMANOVA; *p* > 0.05). Axes 1, 2 and 3 represent the variation percentage that is explained by the tested variable, in this case exercise. Legend: CG pre: control group at pre-intervention (dark blue); CG post: control group at post-intervention (light blue); EG pre: exercise group at pre-intervention (dark orange); EG post: exercise group at post-intervention (light orange); S(number) in the figures (**C**) and (**D**) refers to each subject.

Regarding β-diversity, both weighted and unweighted UniFrac indices did not show significant differences between pre- and post-intervention within study Groups (Supplemental Table S5 and Figure S3; PERMANOVA, *p* > 0.05). PCoA plots from UniFrac distances and Bray–Curtis dissimilarity matrix at genus level did not reveal either clusters by Group (Control or Exercise) or by Time (Pre- versus Post-intervention) (Figure 3A,B). Figure 3C,D illustrate the intrapersonal changes in the gut bacteria community structure in relation to the spatial variation for each Group at the two time points; however, no significant difference was detected. Hence, it seems that subjects had relatively heterogenous responses and it was not possible to observe clustering as an effect of exercise (Figure 3). In view of the possible influence of other variables on β-diversity indices, we applied PERMANOVA considering data from dietary intake, BMI and VO_2_peak; however, there was found no significant difference or clustering among subjects (Supplemental Figure S4; *p* > 0.05).

### 3.3. VO_2_peak and BMI Are Associated with Gut Bacteria Composition

**Figure 4:**
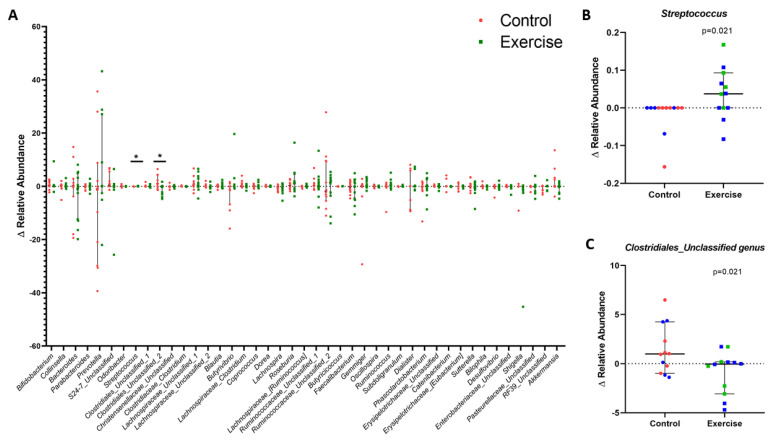
Intragroup variations revealed significant differences between Groups. (**A**) Median and CI95% for genera detected in ≥25% of samples. Asterisk means a significant difference between Groups (*p* > 0.05). (**B**) Median and CI95% for *Streptococcus* and (**C**) Mean with CI95% for the genus of *Clostridiales* order. Each point refers to the delta (∆) of one subject. Red spheres are Control subjects while green squares are subjects from Exercise Group. Blue points are individuals with BMI > 25 kg/m^2^.

Next, we investigated the differences in OTU relative abundance. In our population, a total of 10 phyla were detected of which Bacteroidetes, Firmicutes and Proteobacteria were the three most abundant (Supplemental Table S6). Although both Groups were similar in the main physiological variables at the baseline, high interindividual variations were observed at the phylum level and no significant difference was observed between Groups or within groups between pre- and post-intervention (Supplemental Figure S5; *p* > 0.05). At the genus level, statistical analysis revealed that CG and EG diverged over time in abundance of the *Streptococcus* genus (non-parametric *p* = 0.021; CI95% = 0–0.105; Hodges-Lehmann *p* = 0.047) and an unclassified genus belonging to *Clostridiales* order (parametric *p* = 0.021; CI95% = −4.24–0.313; F = 0.612; Supplemental Table S7). It was observed that EG subjects had an increase in the mean relative abundance of *Streptococcus* genus in parallel with a decrease in that of one *Clostridiales*-order genus after the training period (Figure 4).

**Figure 5:**
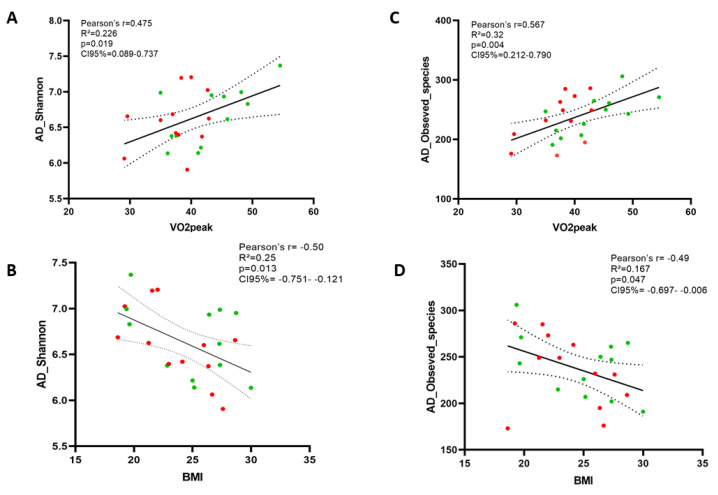
Oxygen consumption (VO_2_peak) and Body Mass Index (BMI) were associated with gut bacteria richness. Figures (**A**) and (**C**) present associations with Shannon index and figures (**B**) and (**D**) show associations with Observed_species. Red points in the graph refer to control subjects while green points represent exercise subjects.

**Figure 6:**
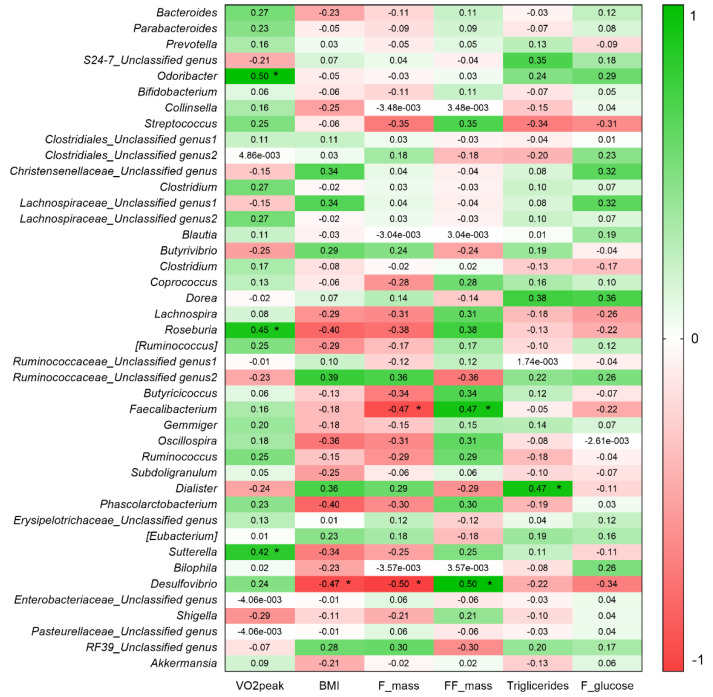
Association between physiological variables and relative abundance of genera (%). Pearson’s R coefficient or Spearman’s partial correlation coefficient were plotted inside of each square in the above figure. The asterisk inside a square indicates significant association between the two variables (*p* < 0.05). Correlations were performed with post-intervention data.

We also performed the ANCOM test, which is a reasonable methodology based on compositional log-ratios that accounts for constraints to reduce inaccuracies in detecting differences in microbial mean taxa abundance. At the species level, ANCOM did not show significant differences between Groups or within groups between pre- and post-intervention (Supplemental Table S8). Finally, we searched for associations between exploratory variables and bacterial composition data in order to investigate whether potential confounding variables were associated with microbiota outcomes. As we expected, VO_2_peak positively correlated with Alpha diversity indices and genera relative abundance, while BMI negatively correlated with these outcomes (Figure 5 and Figure 6).

Specifically, VO_2_peak was positively associated with *Odoribacter* (rho Spearman = 0.505; *p* = 0.012; 95%CI = 0.115–0.760), *Roseburia* (rho Spearman = 0.453; *p* = 0.026; 95%CI = 0.048–0.730) and *Sutterella* (rho Spearman = 0.417; *p* = 0.043; 95%CI = 0.003–0.708) relative abundances, while BMI was associated with *Desulfovibrio* (rho Spearman = −0.472; *p* = 0.02; 95%CI = −0.741–0.072). A negative association was also observed between *Faecalibacterium* and body fat (Pearson’s r = −0.466; R^2^ = 0.217; *p* = 0.022; 95%CI = −0.732–0.077) and a positive association between this genus and free fat mass percentage (Pearson’s r = 0.466; R^2^ = 0.217; *p* = 0.022; 95%CI = 0.077–0.732) (Figure 6).

From baseline data of food habits (Supplemental Figure S6), associations were found between both carbohydrate (Spearman’s r = 0.44. *p* = 0.03; 95%CI = 0.035–0.724) and protein intake (Spearman’s r = −0.56; *p* = 0.004; 95%CI = −0.793–0.197) with Observed_species, between consumption of cereals, bread and tubers with both *Firmicutes* relative abundance (Pearson’s r = −0.415; R^2^ = 0.172; *p* = 0.043; 95%CI = −0.70–0.014) and F/B ratio (Pearson’s r = 0.413; R^2^ = 0.17; *p* = 0.045; 95%CI = 0.011–0.699), and between *Proteobacteria* phylum with mean daily fruit consumption (Spearman’s r = 0.428; *p* = 0.037; 95%CI = 0.0169–0.715). Fasting glucose and triglycerides also correlated with the relative abundance of *Bacteroidetes* and *Verrucomicrobia* phyla, respectively (Supplemental Figure S7).

## 4. Discussion

Moderate aerobic exercise is known to improve physical health, mainly through the cardiorespiratory system, which affects energetic metabolism, neuronal and hormonal activities and immune tolerance and thus can affect gut microbiota [6,14,18]. In our study, EG subjects experienced substantial improvement of cardiorespiratory fitness parameters (measured by VO_2_peak, peak power, first aerobic threshold; respiratory exchange ratio), which was not observed in CG. To the best of our knowledge, this is the first controlled and randomized study design in a homogenous population with an aerobic exercise intervention capable of promoting significant cardiorespiratory improvements without changing gut-affecting variables, such as body composition, food habits or metabolic parameters. Thus, it allowed us to better visualize the effect of exercise on gut bacteria composition. Our findings highlight a regulator effect of moderate aerobic exercise associated with cardiorespiratory fitness improvement and body composition status.

Compared to previous studies with adult populations [10,27,45,46,47], our subjects from both groups presented high alpha diversity (Supplemental Table S4), which is an important metric for evaluation of dysbiosis. Gut bacteria diversity is commonly analyzed in microbial studies as one of the main parameters reflecting the stability, balance, resilience and/or predominance among species. This is important because a low diversity favors dysbiosis [10,48] which, in turn, is associated with Noncommunicable Diseases (NCD) [7,10,45,48]. After 10 weeks of aerobic moderate exercise training, no significant change in α-diversity was observed. Likewise, Allen et al. [24], Cronin et al. [25] and Munuka et al. [26] did not find significant alterations in α-diversity indices after a period of moderate aerobic exercise in an adult population. Six months of leisure-time exercise at moderate intensity had a subtle effect on α- and β-diversity [47], and we observed that Kern’s population had lower α-diversity than our sample [47]. According to our β-diversity PCoA plots, we observed heterogenous responses among individuals and, according to Sket et al. [12,13], our control group of sedentary people may not have changed in a healthy way. Moreover, considering that gut microbiota is affected by genetics and several environmental factors, it is hypothesized that α- and β-diversity changes require a greater and/or longer stimulus. Bycura et al. [49] have proposed that differences between active or athletic and non-active individuals observed in cross-sectional studies may be the result of long-term lifestyle influences and training levels. Moreover, similar studies to ours have failed to include a Control Group, which directly influences results’ interpretation [24,26,49].

Our microbiome analyses revealed no significant changes at the phylum, class, order, family and species levels. In our population, the phylum Bacteroidetes was more abundant than Firmicutes, which seems to be in accordance with Brazilian food habits with daily predominance of carbohydrates and glycan sources [50]. Interestingly, a median reduction of 47.67% was observed in the Proteobacteria phylum of EG subjects, which was not observed in the CG, and we speculate that this may be a regulation caused by moderate aerobic exercise (Supplemental Table S6; *p* > 0.05). This observation is relevant since the pathogenicity of a microorganism can be linked to balance among microbes and their interaction with the host, along with its own abundance [22,51]. In congruence with our perspective, Castellanos et al. [52] identified key bacteria driving the transition of a sedentary to an active lifestyle and vice versa independent of bacterial abundances. The authors highlighted that these two conditions involved a reorganization of an unclassified genus from *Clostridiales* order, unclassified taxa from *Streptococcus* genus and species from *Sutterella*, *Roseburia* and *Odoribacter* genera, all which is in accordance with our findings. These outcomes from Castellanos’ study were observed in a similar population of young healthy adults, although it was an observational design [52]. In human gut microbiota, *Clostridiales* order is composed of a high variety of commensals and opportunistic species while *Streptococcus* genus contains a probiotic species named *S. thermophilus* and commensal species such as *S. salivarius* and *S. vestibularis*. In particular, this latter genus is involved in urea metabolism and lactic acid production, and its metabolites may favor a pH environment that promotes commensal bacteria survival [53]. In agreement with our discussion, several authors have suggested that Clostridia and *Streptococcus* may be potential drivers of population dynamics in the intestine, capable of influencing microorganism interactions and gut homeostasis [52,54,55]. Moreover, a recent observational study by Galle et al. found both *Clostridia* taxa and *Streptococcus* to be abundant among different BMI and physical activity levels, which highlights the importance of these bacteria in the gut ecosystem [56].

The VO_2_peak from EG subjects was positively associated with Alpha diversity indices, as has also been observed in previous studies [27,49,57]. Positive associations were also observed between VO_2_peak and the relative abundances of *Roseburia*, *Odoribacter* and *Sutterella*. Bacteria from *Roseburia* genus are known to produce short-chain fatty acids and may selectively favor key members of the gut microbiota [58]. Estaki et al. [27] and Bressa et al. [59] also claimed that *Roseburia* is related to better physical condition. On the other hand, *Odoribacter* and *Sutterella* genera are often associated with disease conditions [60,61,62,63]; however, one interesting study with a polyphenol intervention in healthy adults showed an increase of *Sutterella* [64] and a resveratrol treatment in diabetic mice also improved *Odoribacter* abundance [65]. In summary, these key bacteria seem to be associated with antioxidant defenses and thus may be responsive to exercise training effects.

Body composition is also a health metric that can be improved by moderate aerobic exercise [5]. Although EG subjects’ BMI, food habits or blood parameters did not significantly change after 10 weeks of exercise training, it was possible to observe a tendency towards losing body fat and gaining free fat mass as an effect of exercise, which did not occur with CG (Table 1). Thus, corroborating the literature [15,45], the mutual influence between both gut bacteria diversity and genus and body composition on the EG subjects may reflect the energy metabolism adaptations caused by exercise. Moreover, *Faecalibacterium* genus and body composition were correlated, and this genus has been suggested as a biomarker for obesity and inflammatory status [66]. Therefore, our correlations suggest that, aside from VO_2_peak, the exercise effect on gut microbiota may depend on BMI status, in concordance with Allen et al. [24]. Finally, in agreement with Moitinho-Silva et al. [67] who also performed a randomized exercise intervention in sedentary adults, the transition from a sedentary to an active lifestyle may lead to heterogeneous adaptations in order to regulate what could be unbalanced. Thus, the exercise effect is specific to an individual’s characteristics, genetics and lifestyle [49]. This explains the high divergence among studies [15] and may also justify our correlations observed between phyla and Alpha diversity with dietary information. As discussed by Dorelli et al. [6] and Aya et al. [15], the majority of studies do not control for diet, or are cross-sectional studies comparing athletes and sedentary people or studies with dietary intervention. In our study we applied qualitative and quantitative methods to better characterize and monitor food habits of both EG and CG subjects, as we had instructed the volunteers to maintain their food habits. Still, carbohydrate and protein intake were correlated with alpha diversity and Firmicutes phyla and F/B ratio were correlated with consumption of foods from the group of bread, cereals and tubers, which is also a source of carbohydrate and protein intake [33]. Daily consumption of fruits also provided a source of carbohydrates [33]. Thus, we suggest that carbohydrates and proteins are important nutrients related to gut bacteria composition, and future comparisons with our study should consider our population’s food habits, as well as sex and age range.

Lastly, our major goal was to evaluate the effect of a structured exercise protocol in a real-world scenario, applying the minimum exercise requirements from WHO recommendations to maintain health status. Our study findings have external validity and our exercise intervention demonstrated efficiency in improving cardiorespiratory fitness. Another important strength of our work was the maintenance of volunteers’ lifestyle and metabolic parameters throughout the study, as well as minimal body mass index changes, which reduced the influence of these variables on our observed gut microbiota variations. Furthermore, we carried out 16S rRNA gene sequencing, V4 region amplification, and bioinformatics steps in similar manner as previous studies in order to enhance comparisons [6,15]. In spite of that, we noticed that even in a homogenous population with very similar lifestyle habits, gut microbiota composition showed high variability. Thus, longitudinal studies with larger samples or in a crossover design may overcome our limitations and provide reliable and applicable evidence in this growing field of exercise and gut microbiome. Moreover, we tested only an aerobic exercise protocol at moderate intensity. Kern et al. [47], Bycura et al. [49] and Moitinho-Silva et al. [67] compared different exercise protocols and demonstrated that they have different effects on gut microbiota composition. Vigorous exercise and resistance training seem to promote different gut microbiota alterations due to their stimulation of metabolic pathways that differently affect the gut system, and this may be of major importance for populations, such as elderly [15,18,68]. We emphasize that it was not our goal to compare different exercise protocols and our outcomes should be carefully interpreted for sedentary men without disease undergoing moderate aerobic exercise. The vast characterization and understanding of exercise effects on the human gut microbiota will be relevant for predicting health risks, as exercise may lead to up- or downregulation of key bacteria related to diseases.

## 5. Conclusions

Our results suggest that 10 weeks of supervised aerobic exercise at moderate intensity was able to increase the VO_2_peak which, along with BMI, showed association with gut bacteria composition in previously sedentary, young, non-obese men who had maintained a balanced consumption of nutrients. The effect of moderate aerobic exercise may depend on a stimulus period able to promote physiological improvements, such as cardiorespiratory fitness and body composition changes, which are related to gut bacteria structure.

## Figures and Tables

**Table 1:** Clinical, anthropometric and cardiorespiratory fitness variables before and after 10 weeks of control and exercise periods.

	PRE			POST		
	CG (*n* = 12)	EG (*n* = 12)	*p* *	CG (*n* = 12)	EG (*n* = 12)	*p* ^#^
Age (years)	25.5 ± 4.66	25.58 ± 5.07	0.96	-	-	-
Weight (kg)	72.15 ± 10.99	77.36 ± 13.19	0.30	72.39 ± 11.17	76.52 ± 12.78	0.26
BMI (kg/m^2^)	23.68 ± 3.29	25.28 ± 4.11	0.30	23.75 ± 3.31	24.90 ± 3.69	0.69
%FM	21.87 ± 12.18	23.59 ± 11.63	0.72	21.85 ± 12.08	21.65 ± 9.32	0.77
%FFM	78.12 ± 12.18	76.40 ± 11.63	0.72	78.14 ± 12.08	78.35 ± 9.32	0.77
Triglycerides (mg/dL)	103.42 ± 11.24	100.45 ± 15.45	0.59	106.30 ± 17.93	99.41 ± 12.18	0.64
Total chol. (mg/dL)	172.34 ± 22.77	166.10 ± 11.00	0.40	165.80 ± 22.10	157.26 ± 15.46	0.83
HDL (mg/dL)	55.71 ± 9.75	54.70 ± 5.74	0.76	68.70 ± 15.33	71.16 ± 8.80	0.57
LDL (mg/dL)	88.11 ± 22.11	86.29 ± 34.65	0.87	93.01 ± 30.03	89.67 ± 45.69	0.93
Fasting glucose (mg/dL)	92.02 ± 8.94	93.95 ± 8.82	0.26	92.51 ± 8.34	91.78 ± 12.38	0.43
VO_2peak_ (ml·kg^−1^·min^−1^)	37.37 ± 4.68	35.83 ± 7.68	0.56	37.60 ± 4.52	42.90 ± 6.00	<0.001
W_peak_ (watts)	297.31 ± 45.14	288.22 ± 34.18	0.56	297.68 ± 46.50	360.27 ± 52.09	0.001
AT1 (seconds)	498 ± 92.77	502.16 ± 111.96	0.88	485.72 ± 132.01	634.33 ± 124.92	0.01
RER	1.30 ± 0.06	1.34 ± 0.16	0.52	1.35 ± 0.07	1.26 ± 0.10	0.03
HR_peak_ (bpm)	188.33 ± 8.46	186.66 ± 9.35	0.65	184.54 ± 9.40	183.83 ± 8.49	0.85

Data are presented as mean ± standard deviation. * The *p*-value refers to the main effect of the Group at the pre intervention time measured by Student’s *t*-test with Welch’s correction. ^#^ The *p*-value refers to the Group × Time interaction measured by two-way ANOVA with repeated measures. Legend: CG: control group; EG: exercise group; PRE: pre-intervention; POST; post-intervention; BMI: body mass index; % FM: fat mass percentage; % FFM: fat mass percentage; CHOL.: cholesterol; HDL: high density lipoprotein-cholesterol; LDL: low density lipoprotein-cholesterol; mg/dL: milligrams per deciliter of plasma; Wpeak: peak power in watts; VO_2_peak: peak oxygen consumption; AT1: first aerobic threshold; RER: respiratory exchange ratio; HRpeak: maximum heart rate in beats per minute (bpm).

## Data Availability

The raw data (FASTQ format) are available from the National Center for Biotechonology Information (NCBI) Biosample database (https://www.ncbi.nlm.nih.gov/bioproject/PRJNA592775/ (accessed on 1 December 2019)) with accession number PRJNA592775.

## Supplementary Materials

The following are available online at https://www.mdpi.com/article/10.3390/nu13082839/s1, Figure S1: Individual variations in the cardiorespiratory fitness of the Control and Exercise Groups, Figure S2: Alpha Diversity indices, Figure S3: Group significance plots from Unweighted UniFrac index, Figure S4: Principal coordinates analysis (PCoA) of gut microbiota composition based on the Body Mass Index (BMI) and Peak Oxygen Consumption (VO_2_peak), Figure S5: Relative abundance at the phylum level and among all subjects (CG = 12; EG = 12) at the pre- and post-intervention, Figure S6: Association between food intake and bacterial composition data, Figure S7: Association between physiological variables and bacterial composition data. Table S1: Participants’ distribution between Groups considering the baseline values of BMI and VO2peak variables, Table S2: Total daily energy and nutrients intake before, during and after 10-week period, Table S3: Frequency of reads per sample and respective metadata, Table S4: Alpha Diversity analysis, Table S5: Variations in the average community structure, Table S6: Relative abundance from all detected Phyla, and from Family and Genera presented in at least 50% of samples, Table S7: Intragroup variations at the Genus level, Table S8: ANCOM results from species level.
