# Supplementary material for: Changes in the Gut Bacteria Composition of Healthy Men with the Same Nutritional Profile Undergoing 10-Week Aerobic Exercise Training: A Randomized Controlled Trial
Source: Nutrients. 2021 Aug 18;13(8):2839. doi: 10.3390/nu13082839 (PMC8398245; doi:10.3390/nu13082839)
Supplement: Supplementary file 1 [file nutrients-13-02839-s001.zip › nutrients-1292612-supplementary/nutrients-1292612-supplementary.pdf]

SUPPLEMENTAL FIGURES

Changes in the Gut Bacteria Composition of Healthy Men with the same Nutritional Profile Undergoing 10-week Aerobic Exercise Training: A Randomized Controlled Trial

Ayane S. Resende [1], Geovana S. F. Leite [2] and Antonio H. Lancha Junior [3,*]

[1] Health Sciences Graduate Program, Federal University of Sergipe, Sao Cristovao 49100-000, SE, Brazil; ayanesa@academico.ufs.br

[2] Laboratory of Nutrition and Metabolism Applied to Motor Activity, School of Physical Education and Sports, University of Sao Paulo, Sao Paulo 05508-030, SP, Brazil; geovana.leite@usp.br

[3] Laboratory of Clinical Investigation: Experimental Surgery (LIM/26), Clinics' Hospital of Medical School, University of Sao Paulo, 01246-903, SP, Brazil.

* Correspondence: lanchajr@usp.br; Tel.: +55-11-3061-7474.

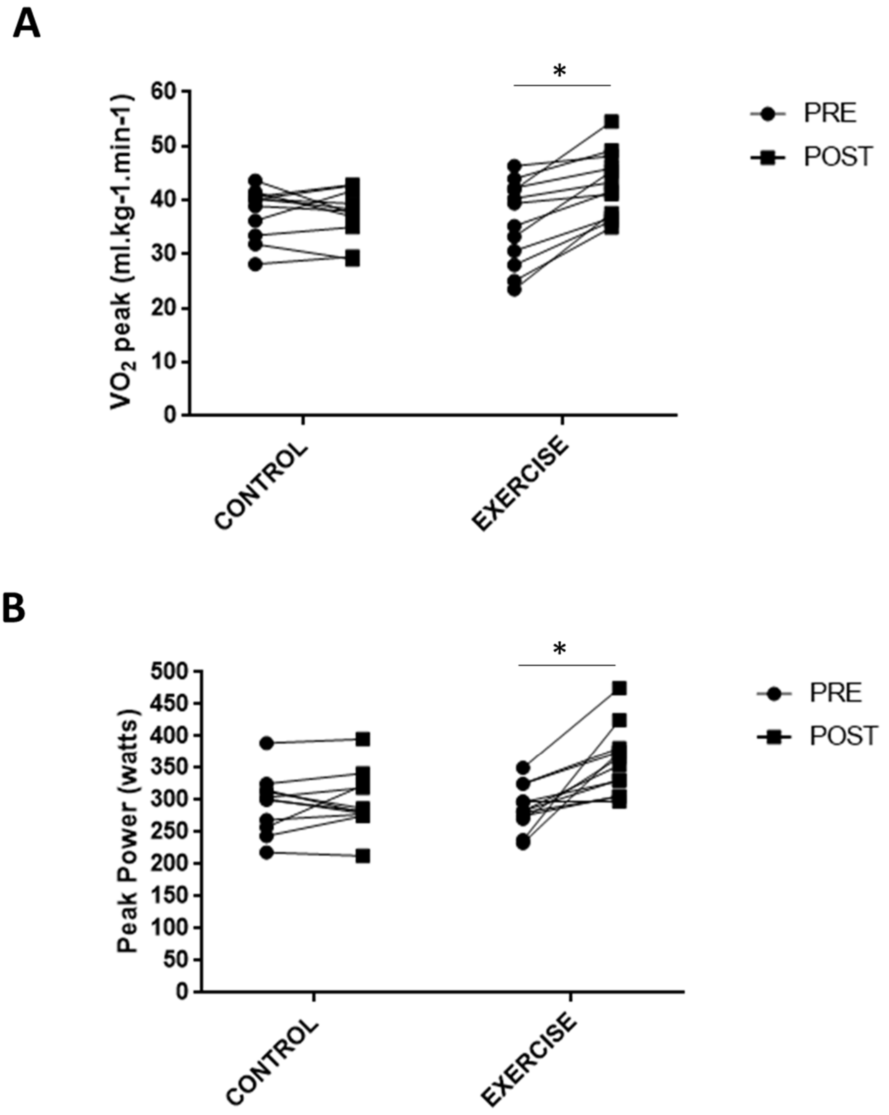

Figure S1. Individual variations in the cardiorespiratory fitness of the Control and Exercise Groups. **(A)** Oxygen consumption and **(B)** potency variables obtained from cardiorespiratory fitness test. *p<0.05 (Group x Time interaction by ANOVA two way with repeated measures). **Legend:** PRE: pre-intervention; POST: post-intervention.

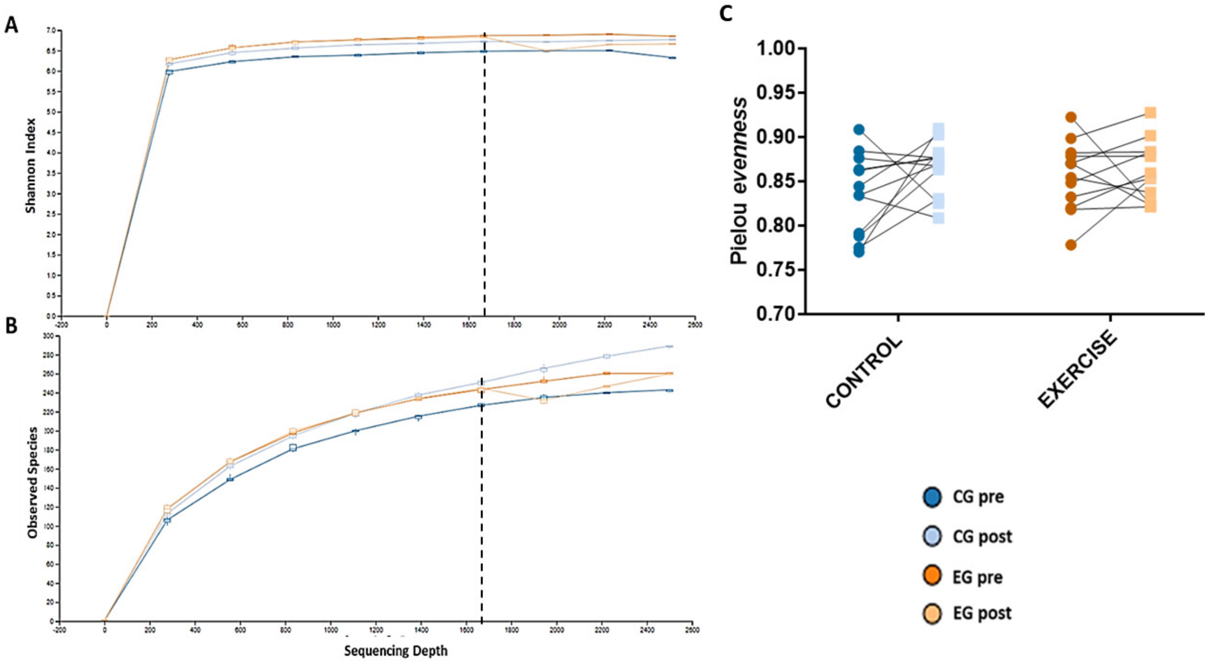

Figure S2. Alpha Diversity indices. Rarefaction curves presented by **(A)** Shannon index and **(B)** observed species show saturation with 1,667 sequences per sample (dotted line; n = 48 samples). Each colored line connects a mean number and standard deviation corresponding to 97% similarity between the OTUs observed for each Group and Time. **(C)** Individual variations of the Control and Exercise Groups according to Evenness index. **Legend:** CG pre: control group at pre-intervention (dark blue); CG post: control group at post-intervention (light blue); EG pre: exercise group at pre-intervention (dark orange); EG post: exercise group at post-intervention (light orange). $p > 0.05$ by two-way ANOVA test with repeated measures.

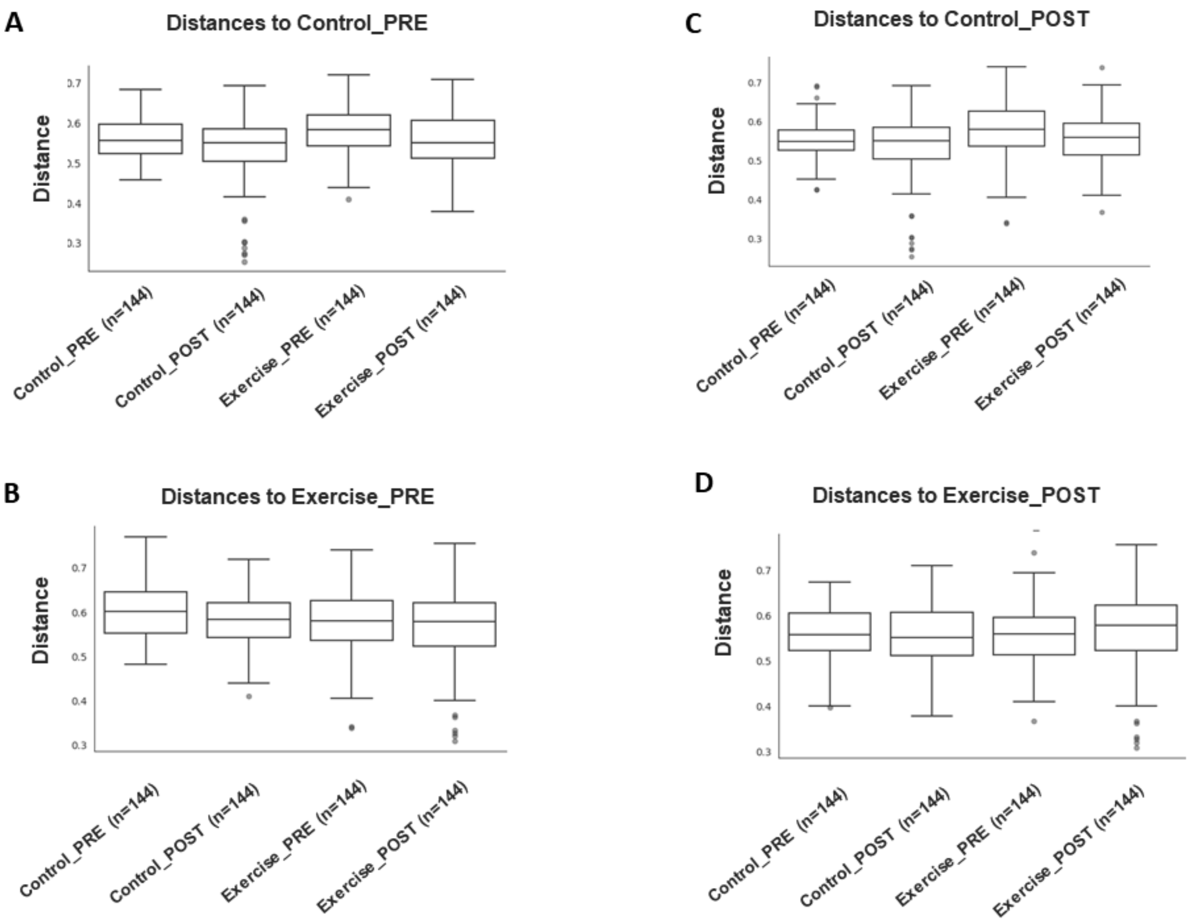

Figura S3. Group significance plots from Unweighted UniFrac distance (PERMANOVA). Statistical analysis related to this figure is shown in Supplemental Table S5. **Legend:** PRE: pre-intervention; POST: post-intervention.

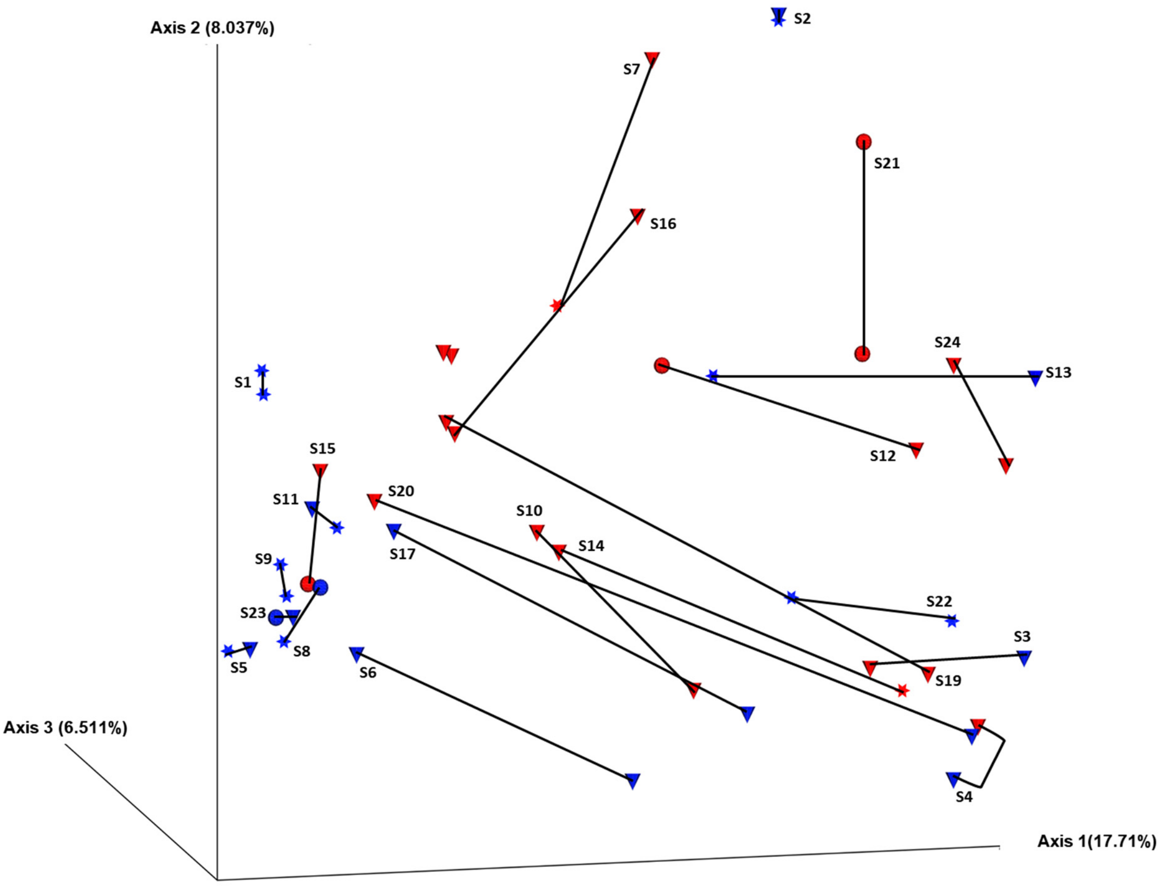

Figure S4. Principal coordinates analysis (PCoA) of Gut Microbiota composition based on the Body Mass Index (BMI) and Peak Oxygen Consumption (VO_2peak). This PCoA plot was constructed from Bray-Curtis dissimilarity matrix at the Genus level, which was based on the 1,667 microbial sequences from the rarefaction curve. Each point in the PCoA plot represents one individual, which is colored according to its BMI classification. The red color refers to individuals with normal BMI and the blue color refers to overweight subjects. The lines between two points denotes the intraindividual spatial variation over 10-week period, linking the same individual from its baseline to the post-intervention. The shape of the points symbolizes the categorization of VO_2peak of the subjects. Stars ( ) refers to volunteers with low VO_2peak, cone ( ) are subjects with an average VO_2peak and spheres ( ) are the ones with high VO_2peak. There was no difference between pre- to post- intervention in neither Group (PERMANOVA; p>0.05). Axes 1, 2 and 3 represent the variation percentage that are explained by each coordinate. **Legend:** S(number) refers to each subject that participated in this study from a total of 24 subjects.

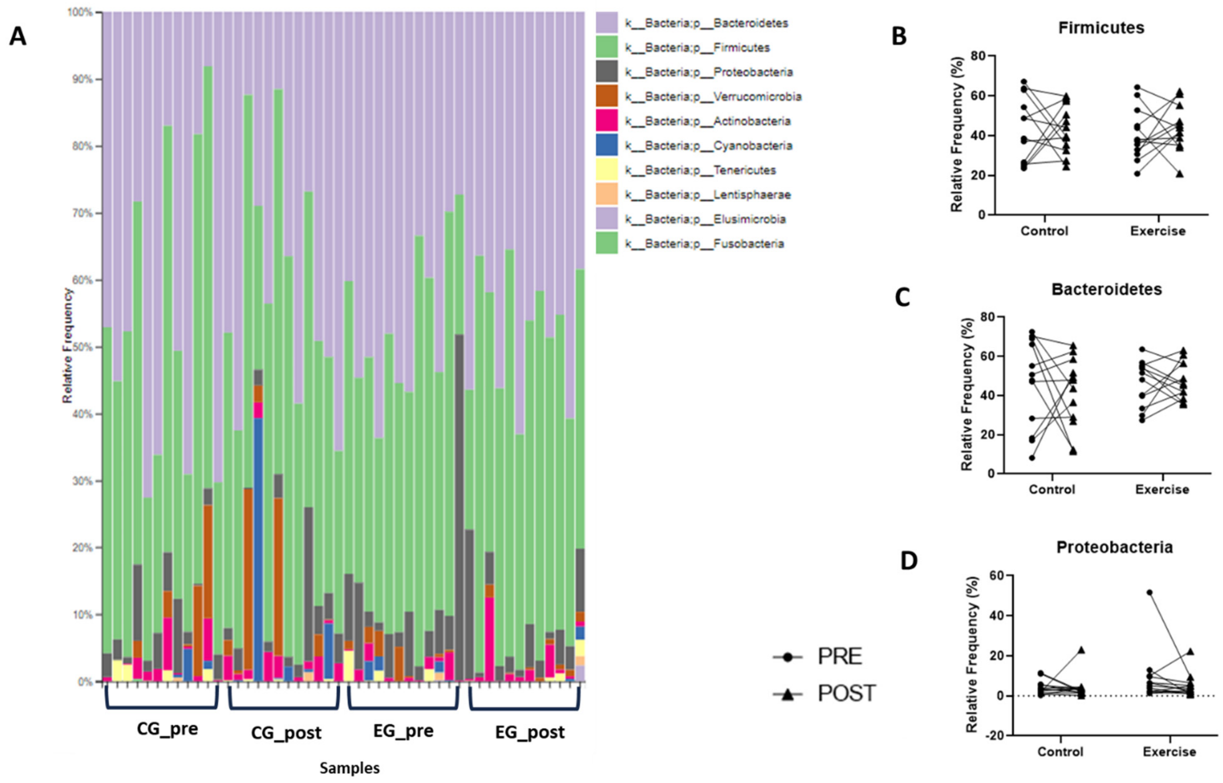

Figure S5. Relative abundance at the Phylum level and among all subjects (CG = 12; EG = 12) at the pre- and post-intervention. **(A)** Each histogram bar refers to a volunteer of a specific Group and Time, which are paired, that is, the first bar of CG_pre is the same volunteer of the first bar of CG_post, and so on. **(B, C and D)** Individual variations of the most abundant Phyla from the pre- to the post-intervention. **Legend:** CG_pre: control group at pre-intervention; CG_post: control group at post-intervention; EG_pre: exercise group at pre-intervention; EG_post: exercise group at post-intervention; PRE: pre-intervention; POST: post-intervention.

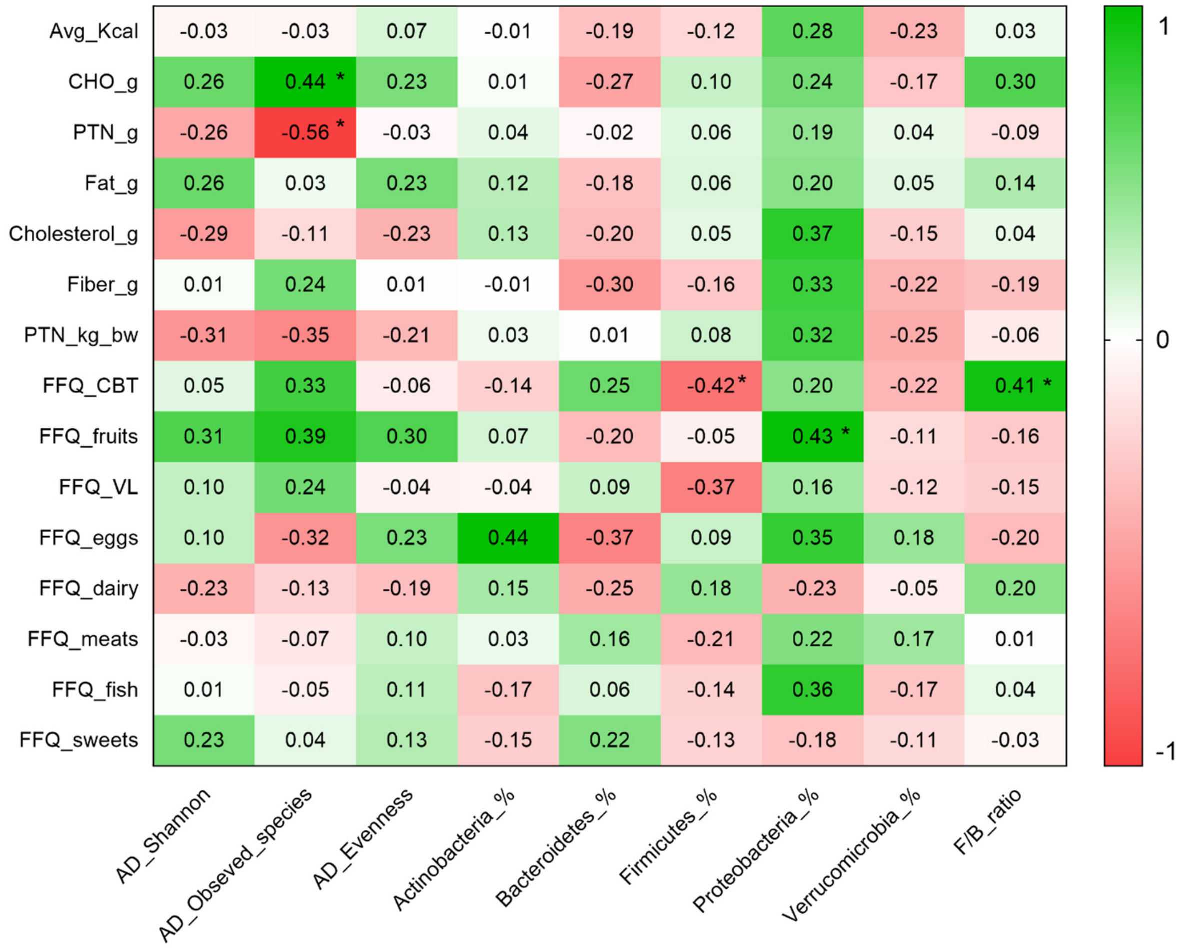

Figure S6. Association between food intake and bacterial composition data. According to the normality of the data, Pearson's R coefficients or Spearman's partial correlation coefficients were plotted inside of the table. The asterisk inside of the square indicates significant association between the two variables ($p<0.05$). Correlations were performed with data from the baseline. **Legend:** Avg_Kcal: Mean energy intake; _g: grams; CHO; carbohydrate; PTN: protein; PTN_kg_bw: protein consumption per kilograms of body weight; FFQ: food frequency questionnaire; CBT: cereals, bread and tubers; VL: vegetable and legumes; AD: alpha diversity; %: relative abundance; F/B ratio: Firmicutes and Bacteroidetes ratio.

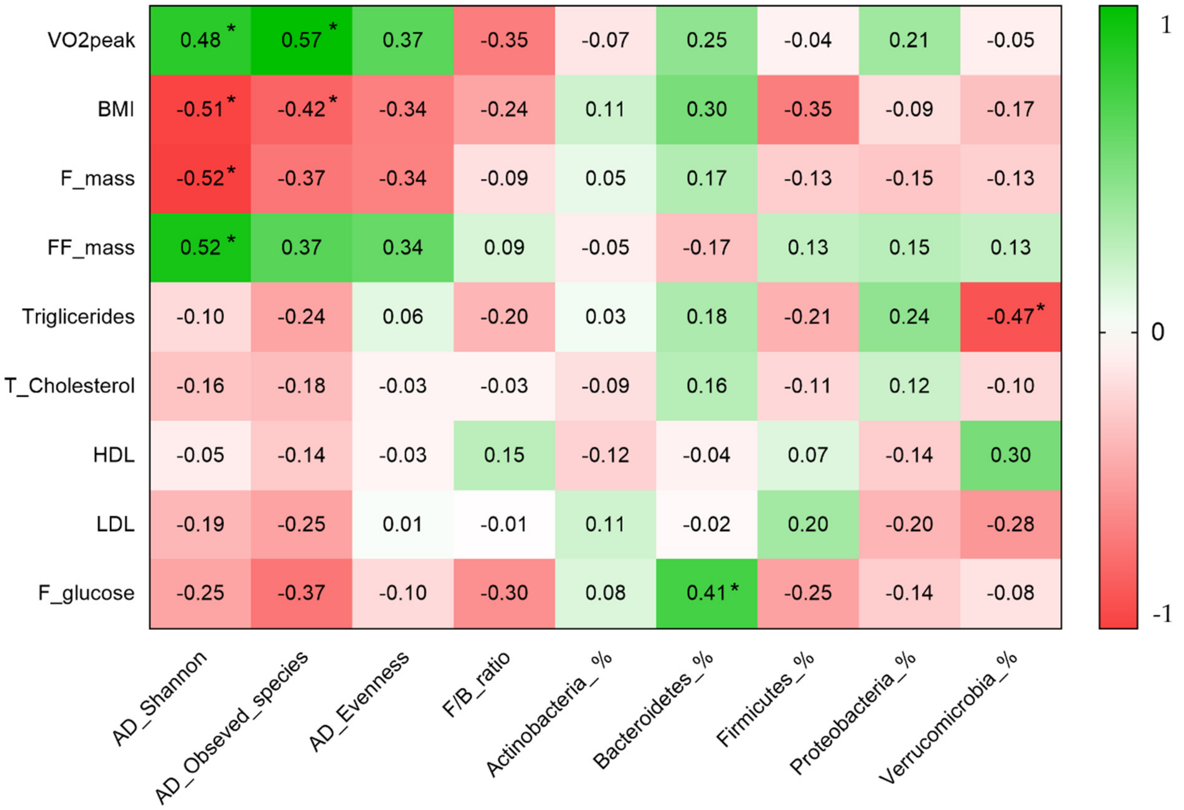

Figura S7. Association between physiological variables and bacterial composition data. According to the normality of the data, Pearson's R coefficients or Spearman's partial correlation coefficients were plotted inside of the table. The asterisk inside of the square indicates significant association between the two variables ($p<0.05$). Correlations were performed with data from the post intervention. **Legend:** VO2: peak oxygen consumption; BMI: body mass index; F_mas: fat mass (%); FF_mass: free fat mass (%); T_cholesterol: total cholesterol from blood samples; HDL: high density lipoprotein-cholesterol; LDL: low density lipoprotein-cholesterol; F_glucose: fasting glucose from blood samples; AD: alpha diversity; F/B ratio: Firmicutes and Bacteroidetes ratio; %: relative abundance;
